# A comparative study examining the perspectives of both students and clinical teachers during practical teaching sessions in otorhinolaryngology

**DOI:** 10.1186/s13104-026-07802-w

**Published:** 2026-04-27

**Authors:** Otto-Alexander Hofmann, Christian Offergeld, Wolf Ramackers

**Affiliations:** 1 Department of Otorhinolaryngology, Head and Neck Surgery, Medical Center - University of Freiburg, Freiburg, Germany; 2 Department of General, Visceral, Pediatric and Transplant Surgery, University Hospital RWTH Aachen, Aachen, Germany; 3 Department of Medical Education, Hannover Medical School, Hannover, Germany

**Keywords:** Medical education, Otolaryngology training, Student-teacher interaction, Teaching evaluation

## Abstract

**Objective:**

Medical education aims to prepare future physicians for high-quality patient care. This prospective study examines the perceptions of medical students and clinical teachers with regard to teaching in otorhinolaryngology (ENT) clinical rotations. The objective is to identify discrepancies between the perceptions of students and clinical teachers and their potential impact on overall satisfaction with the learning and teaching process.

**Methods:**

During a two-week ENT rotation, medical students were obliged to attend seven different seminars. At the end of each seminar, both the students and their teachers were asked to evaluate the seminar using a standardized questionnaire. A total of 1139 questionnaires (*n* = 1089 students and *n* = 50 teachers) were collected. Data analysis included a descriptive evaluation and a comparison between the two groups using the Mann-Whitney U test and Cliff’s delta.

**Results:**

The evaluation revealed a positive perception of teaching as measured by high overall scores, with students averaging 13.2 (± 1.7) and teachers 13.4 (± 1.1) out of a maximum grade of 15. Our analysis revealed no statistically significant disparities in the majority of the question items relating to perceptions between students and clinical teachers. Statistically, highly significant differences with relevant effect sizes were only applicable for the question items: *Preparation*,* Participation by Schedule and Understandable Explanations*.

**Discussion:**

Our analysis suggests that there is a high level of agreement between students and teachers in terms of perception and satisfaction. The discrepancies in the areas of *Preparation*, *Participation by Schedule*, and *Understandable Explanations* relate to the nature of student-teacher interactions. This indicates that even when overall perceptions are positive, the interactions between students and clinical teachers may still be viewed differently, highlighting an opportunity for improvement.

**Conclusion:**

Our study shows that medical students and clinical teachers largely share similar perceptions of teaching and learning in practical ENT seminars. Differences were limited to *Preparation*, *Participation by Schedule*, and *Understandable Explanations*. Addressing these discrepancies through improved communication may help optimize the learning environment.

**Supplementary Information:**

The online version contains supplementary material available at 10.1186/s13104-026-07802-w.

## Introduction

Despite its clinical relevance, undergraduate exposure to otorhinolaryngology (ENT) remains limited, although ENT-related presentations are common across multiple disciplines, including primary care and emergency settings [1–4]. Medical students also report insufficient opportunities to acquire practical ENT skills, highlighting the importance of supervised, hands-on training to develop clinical competence and critical thinking [5–7].

A supportive teaching climate, clear communication of learning objectives, and the ability to adapt to student needs are several key factors that can greatly impact student engagement and skill acquisition. Understanding how teaching methods are perceived by students and clinical teachers is of paramount importance, as these perceptions play a significant role in shaping the quality of teaching and learning [8, 9]. For example, students may leave a teaching session with unanswered questions, while clinical instructors may perceive the teaching as successful. This highlights the need to examine differences in perceived learning outcomes between both groups.

Structured assessments facilitate the collection of student feedback on teaching quality and support the identification of strengths and areas for improvement through the analysis of data. Beyond knowledge transmission, clinical teachers are perceived as role models, with non-cognitive characteristics such as approachability and enthusiasm strongly shaping learning, specialty choice, and future teaching aspirations [10, 11].

Comparative evidence on how medical students and clinical teachers perceive educational methods and its environment remains limited, as prior research has primarily examined both groups in isolation [8, 10, 12–14].

This prospective study aims to explore whether medical students and clinical teachers hold similar or different perceptions regarding practical sessions in the ENT department. Its goal is to provide valuable insights for clinical teachers, faculty and ENT Societies to provide data for improving the training of medical educators in the ENT departments.

## Methods

In this single-centre, prospective study conducted at the ENT Department of the University Hospital Freiburg, Germany during the summer semester of 2023, 172 medical students in their 7th or 8th semester and 17 clinical teachers were involved. The ENT course, integral to the regular academic curriculum, was structured as a two-week block, conducted in seven sequential groups with 23 to 28 participants in each group. This ensured comprehensive ENT coverage via the following seven clinical skills: sessions on mirror examination, oncology, laryngology, rhinology, otology, emergencies, and communicative competencies. To facilitate preparatory and concurrent learning, an e-learning module was accessible via the clinic’s online platform.

To guarantee an unbiased collection of participants and equitable distribution across the ENT block courses, medical students and clinical teachers from the appropriate population at the University Hospital Freiburg were involved, without any intervention or pre-selection by the research group.

Ethical considerations were followed, with approval obtained by the ethical commission of the University Hospital Freiburg (23-1227-S1), securing the integrity of participant engagement.

Data was gathered through anonymous questionnaires (Supplementary File 1). Feedback after class has proven to be an important method for improving the quality of clinical medical education [15]. The questionnaire assessed subjective experiences, perceptions, and satisfaction of students and clinical teachers during practical teaching sessions. Questionnaire items were developed through group discussions involving evaluation experts, medical educators, and students, covering key aspects of the educational environment. (Table 1).

Responses were rated on a 6-point Likert scale (1 = disagreement, 6 = highest agreement). Participants reported gender, provided an overall grade (0–15), and could add free-text comments. Questionnaires lacking an overall grade were excluded, and missing item responses were assigned the lowest score [1].

Descriptive statistics (mean, standard deviation, median, and interquartile range) summarized the data. The Mann-Whitney U test, proved by the Kolmogorov-Smirnov and Shapiro-Wilk tests for non-normality, compared groups. Cliff’s Delta quantified effect sizes, with interpretation based on Romano et al. [16]: negligible (|δ|<0.147), small (0.147<|δ|<0.33), medium (0.33<|δ|<0.474), and large (|δ|>0.474). Comparative analyses were set with *p* < 0.05 as the significance threshold.

Data analyses were conducted using RStudio (Version 2023.09.1 + 494), with tables created in Excel and Word (Microsoft 2021) to present the findings.

## Results

Of the 1,204 student questionnaires distributed, 1,089 were completed and returned. All 50 of the faculty questionnaires were returned completed, resulting in response rates of 90.4% and 100%, respectively. Six student questionnaires were excluded due to a missing final grade, leaving 1083 student questionnaires and all faculty questionnaires for analysis.

Descriptive statistics for teaching events and questionnaire items are presented in Table 1, including mean, standard deviation, and median with interquartile range.

The analysis revealed high overall satisfaction levels with average scores of 13.2 (SD ± 1.7) for students and 13.4 (SD ± 1.1) for faculty members, on a scale of up to 15 points, indicating a substantial positive skew in evaluations from both groups.

For most questions, *no significant differences with relevant effect sizes* in responses between the two groups were observed across all seven practical courses. (Table 1).

Statistically significant differences were observed in specific areas such as *Teaching Materials*, *Preparation*, *Facilities*, *Participation by Schedule*, *Peers*, *Understandable Explanations*, *Learning Advancement*, and *Expression of Questions*, with p-values (*p* < 0.05).

*Preparation*, *Participation by Schedule* and *Understandable Explanations* displayed even highly significant differences (*p* < 0.0001).

Notably, the interquartile range for *Attendance* among students and *Expression of Questions* and *Peers* among clinical faculty was 6. In contrast, *Participation by Schedule* among faculty showed an interquartile range of 2 to 5.

The study distinguished between two variables - *Preparation* and *Participation by Schedule* - based on their highly significant p-values (*p* < 0.0001) and substantial effect sizes (δ -0.603 and δ 0.713, respectively). Conversely, *Understandable Explanations*, while also showing a highly significant p-value (*p* < 0.0001), demonstrated a moderate effect size of δ 0.335.

**Table 1:** Descriptive comparison of students and clinical teachers, including Mann-Whitney U test p-values and Cliff’s Delta to indicate effect sizes

Questionitem	Students (*n*=1083)				Clinical teachers (*n*=50)				*p*-value	Effect size
	MW	SD	Median	IQR	MW	SD	Median	IQR		(Cliffs Delta)
Overall										
Structure	5,5	0,8	6	[5–6]	5,4	0,6	5	[5–6]	0.1663	0,1
Teaching Materials	5,5	0,8	6	[5–6]	5,3	0,7	5	[5–6]	0.0161	0,171
Course Duration	5,4	0,9	6	[5–6]	5,3	0,7	5	[5–6]	0.0992	0,119
Atmosphere	5,6	0,7	6	[5–6]	5,6	0,6	6	[5–6]	0.4135	0,055
Prior Knowledge	5	1	5	[4–6]	4,9	0,9	5	[4–6]	0.2237	0,096
***Preparation***	3,8	1,7	4	[3–5]	5,5	0,8	6	[5–6]	***< 0.0001***	***-0***,***603***
Facilities	5,2	1,2	6	[5–6]	4,9	1,2	5	[5–6]	0.0232	0,174
***Participation by Schedule***	5,5	0,9	6	[5–6]	3,6	1,6	4	[2–5]	***< 0.0001***	***0***,***713***
Peers	5,5	0,9	6	[5–6]	5,9	0,4	6	[6–6]	0.0105	-0,17
Clinical Teachers										
***Understandable Explanations***	5,7	0,6	6	[5–6]	5,4	0,5	5	[5–6]	***< 0.0001***	***0***,***335***
Learning Advancement	5,6	0,7	6	[5–6]	5,4	0,5	5	[5–6]	0.0045	0,194
Motivation (Teachers)	5,6	0,7	6	[5–6]	5,6	0,5	6	[5–6]	0.7251	0,024
Attendance	5,7	0,8	6	[6–6]	5,5	1	6	[5.2–6]	0.1479	0,083
Expression of Questions	5,5	0,8	6	[5–6]	5,8	0,4	6	[6–6]	0.033	-0,151
Students										
Motivation (Students)	5,3	0,9	6	[5–6]	5,3	0,9	6	[5–6]	0.8698	-0,012
Interest	5,2	1	5	[5–6]	5,3	0,7	5	[5–6]	0.9782	0,002
Participation	5,5	0,8	6	[5–6]	5,5	0,7	6	[5–6]	0.4741	0,051
Capturing Attention	4,8	1,2	5	[4–6]	4,9	0,9	5	[4.2–5]	0.8835	-0,012
Practical Skills										
Feedback	5,1	1,2	6	[4–6]	5,1	1,3	5	[5–6]	0.7167	-0,028
Application of Learned Skills	4,6	1,3	5	[4–6]	4,6	1	5	[4–5]	0.6785	0,034
*Interaction*										
Interaction	5,4	0,8	6	[5–6]	5,7	0,5	6	[5–6]	0.0769	-0,129
Overall Grade	13,2	1,7	13	[12–14]	13,4	1,1	13,5	[13–14]	0.7164	-0,03

MW = mean; SD = standard deviation; Median; IQR = interquartile range; Bold italic = highly significant p-values (p < 0.0001) with relevant effect sizes

Due to the limited number of students who identified as “diverse” or did not specify their gender, drawing statistically significant and precise conclusions for these groups may be challenging. (Table 2). Therefore, a brief subgroup analysis was conducted, focusing exclusively on the two binary gender categories, “male” and “female,” within the group of students. To ensure statistically significant conclusions can be drawn, the group of clinical teachers was considered as a whole, without differentiation between males and females.

**Table 2:** Gender distribution among students and clinical teachers

Participants	Students (*n* = 1083)	Clinical Teachers (*n* = 50)	*p*-value¹
**Gender**			<0.001
Male	360	35	
Female	487	15	
Diverse	11	0	
Unknown / Not specified	225	0	

¹Chi-square test for independence

A concise subgroup exploration involving male and female student perceptions with those of clinical teachers revealed minimal disparities between the female and male student subgroups. Consequently, the subsequent discussion will focus on the overall differences observed between students and clinical teachers.

## Discussion

Student feedback plays a key role in improving educational quality by helping educators align teaching strategies with students’ needs and refine instructional methods toward a more learner-centered approach [15, 17, 18].

The collected data is robust and reliable, as illustrated by the high response rates achieved in the questionnaire distribution. Medical students achieved a response rate of 90.4%, while clinical teachers reached 100%, indicating strong engagement from both groups. These rates are higher than typical benchmarks reported in similar research, which advances the credibility of our analysis.

**Fig. 1:**
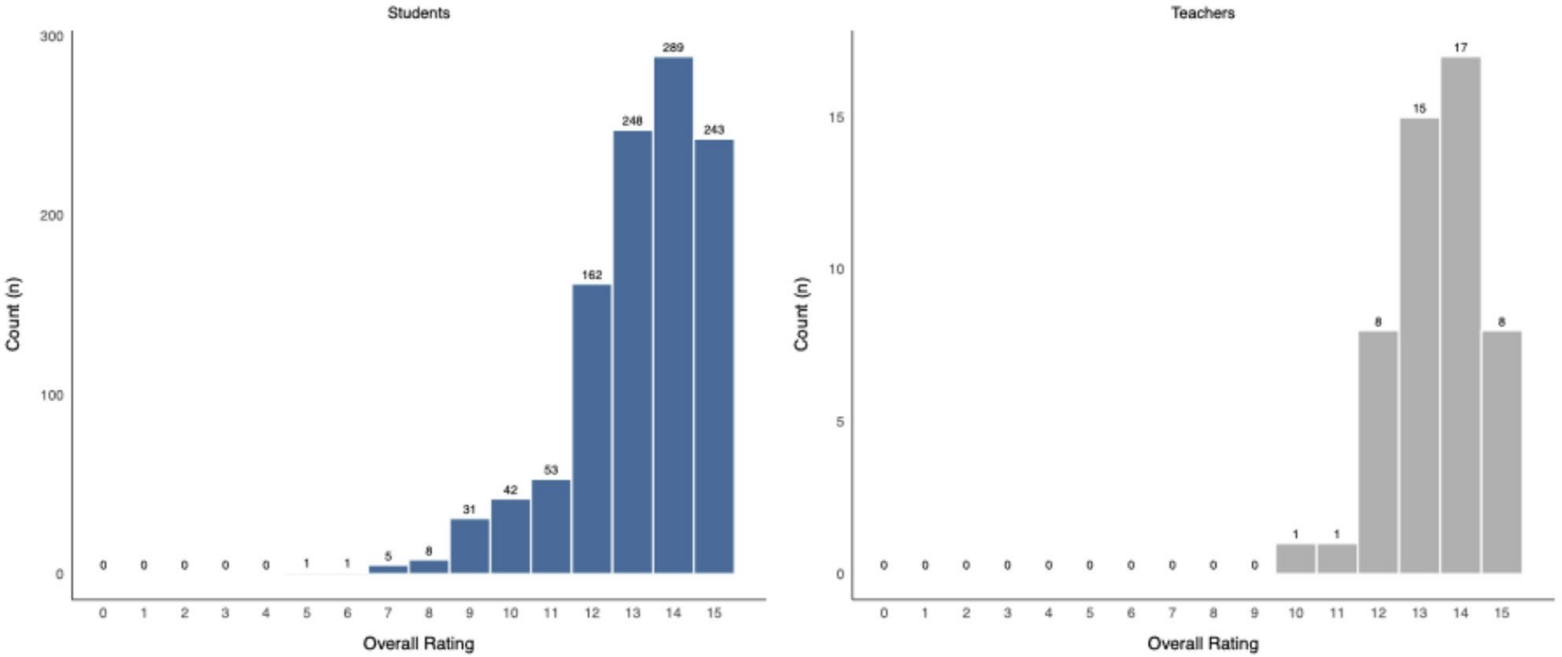
Overall ratings by students and teachers using the German high school grading system (0–15). Bars indicate the number of ratings per grade

The high satisfaction scores of both students (median 13 [12–14]) and clinical teachers (median 13.5 [13–14]) indicate successful teaching practices and a supportive learning environment (Fig. 1). With the exception of *Preparation* among students and *Participation by Schedule* among faculty (both median = 4), the majority of items were rated positively by both groups, with median scores of 5 or higher, reflecting general agreement across assessed dimensions. Motivated and satisfied students and teachers contribute to a mutually reinforcing educational relationship that enhances overall learning quality and outcomes [19, 20].

In our study, most of our question items did not show any statistically significant differences with meaningful effect sizes, except for *Preparation*,* Participation by Schedule* and *Understandable Explanations*.

Despite statistical significance at *p* < 0.05, small effect sizes indicate limited practical relevance [21]. Effect sizes offer more meaningful insight into the magnitude and real-world relevance of differences than p-values alone, particularly in perception-based educational research [22, 23].

Overall, it could be argued that there were *no substantial discrepancies* in the perception of practical teaching sessions between students and clinical teachers, suggesting a shared perspective on the learning and teaching atmosphere.

**Fig. 2:**
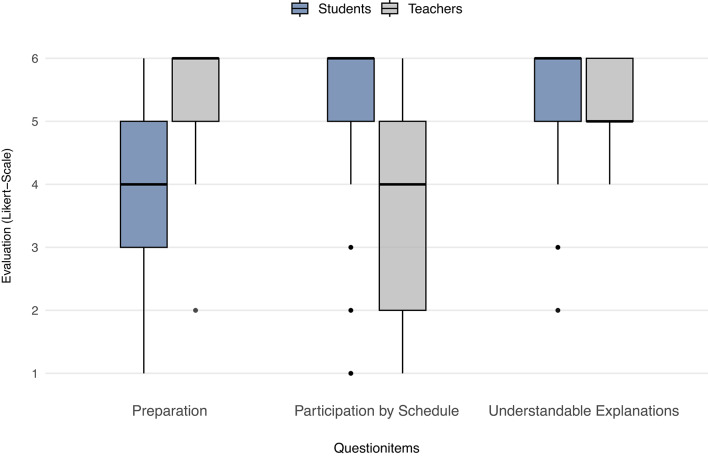
Boxplot of significant differences with relevant effect sizes between students and teachers (6-point Likert scale)

It is noteworthy to highlight the question items that not only showed highly significant differences but also demonstrated moderate to large effect sizes. (Fig. 2).

### Preparation

A significant discrepancy in perceived preparation was observed, with clinical teachers reporting substantially higher levels of preparation than students (δ = −0.603). Previous research suggests that students rely more on external regulation than intrinsic motivation when preparing for coursework, reporting that stricter requirements or assessments would improve preparedness [24]. This underscores the importance of aligning motivational factors with suitable regulatory frameworks.

Digital tools such as e-learning modules can support self-directed learning and individual responsibility and have proven effective as supplements to traditional teaching [25]. Requiring completion of preparatory e-learning modules prior to participation, as described by Kwant et al. [26], may enhance student preparedness and engagement while avoiding excessive pressure.

Clinical teachers’ high preparedness may partly reflect their professional experience, particularly with patient-related content, while didactic preparation was not examined in this study. Prior research suggests that especially junior physicians may feel insufficiently prepared pedagogically [27]. At the same time, variability in student preparedness can limit discussion depth and learning outcomes [28]. The teaching faculty expect a certain degree of foundational knowledge from students, which, if absent, complicates the teaching effort. This challenge is highlighted by a teacher’s remark: “*Overall comment: Students have only minimal prior anatomical knowledge”.*

The importance of preparation is acknowledged mutually by students and clinical teachers, with students perceiving well-prepared teachers as a positive influence on the effectiveness of clinical sessions [12]. This discrepancy may reflect differing expectations, with students adopting a more passive role and expecting teachers to lead seminar activities.

### Participation by schedule

This item examines how student schedules and clinical teachers’ work routines affect participation in teaching sessions. While individual factors such as workload and motivation may influence participation, this analysis focuses on scheduling-related aspects. A clear discrepancy between students and teachers was observed.

Students typically follow structured timetables that allow reliable participation in scheduled sessions. In contrast, clinical teachers must balance teaching with unpredictable clinical demands, including emergencies and ongoing patient care, particularly in hospital settings [29]. Notably, faculty had a wider range of opinions, with an interquartile range of 2–5, suggesting diverse perspectives among the participants.

Despite these constraints, clinical teachers make considerable efforts to prioritize teaching alongside clinical duties, a commitment highly valued by students [30, 31]. Recognizing teaching as an integral component of academic careers may enhance motivation and signal institutional commitment to educational quality.

The challenge of balancing clinical duties with educational responsibilities is underscored not only in various studies pointing to time constraints as a significant barrier to effective clinical teaching, but also through direct comments from our teaching staff, such as: “*Got called 4 times during seminar! Found out 20 minutes before the start of the seminar to take over from a colleague!*” or “*Got paged 8 times; had to make 4 phone calls*” [5, 6, 8, 29, 32].

Optimal engagement in teaching activities extends beyond punctual attendance and initiating courses on schedule; it demands dedicated, uninterrupted time explicitly allocated for clinical teaching.

### Understandable explanations

This item assessed the clarity of teaching as perceived by students and clinical teachers. Clear and comprehensible explanations are a key determinant of effective teaching from the student perspective [8]. In this study, students rated teachers’ explanations more positively than teachers rated themselves. Given the moderate effect size, this difference likely reflects minor variation rather than meaningful disagreement.

This finding may indicate teachers’ reflective and self-critical approach to teaching, as they continuously evaluate and refine their instructional methods. While teachers may question the effectiveness of their explanations, students often perceive them as appropriate for their current level of understanding.

Our findings stress the importance of feedback for teachers to measure the effectiveness of their teaching methods and recognize that their instructional delivery often meets students’ comprehension needs in practical medical courses, despite the observed moderate effect size.

## Limitations

The imbalance between student and teacher sample sizes may be perceived as a limitation when drawing comparative conclusions. The smaller number of clinical teachers warrants more cautious interpretation, as estimates derived from the larger student cohort are inherently more robust. However, this disproportion reflects an intrinsic characteristic of clinical teaching settings. Recognizing this, we collected 50 questionnaires from teachers to gather a substantial amount of faculty data. This approach ensured that we have a robust foundation from which to extract objective insights, diminishing the impact of the uneven ratio and enhancing the validity of our analysis.

Upon careful examination of all questions, it became evident that not all questions could be mirrored precisely between students and faculty. Certain aspects are better suited for either the student or teacher role. For instance, regarding the question item *Teaching Materials*, students were asked whether the teaching material supported their learning, while teachers were asked to assess the adequacy of the study material for the respective class.

Despite these challenges, we were able to address critical aspects of conducting practical teaching sessions and gather perspectives from both students and teachers on key elements such as *Atmosphere*, *Understandable Explanations*, *Learning Advancement* or *Feedback*.

The significant imbalance in gender distribution between students and clinical teachers (*p* < 0.001) (Table 2) represents a potential confounder. While reflecting current demographic structures, this imbalance warrants cautious interpretation of group comparisons and highlights the need for further research.

Given the lack of existing literature on comparing the perceptions of students and clinical teachers, this study provides initial insights and paves the way for further exploration of numerous other variables in future research. We suggest that future studies consider incorporating factors such as the teacher’s level of experience and teaching workload to enrich and replicate the findings of this investigation.

## Implications for education

These findings serve as a starting point, indicating that there is minimal reason to be concerned about a major dichotomy in the expectations and observations of medical students and their clinical teachers during practical seminars in the ENT department. It appears that there is a strong alignment between the perceptions of clinical teachers and students. While minor fluctuations in perspectives may exist, these differences are not substantial enough to lead to a flawed understanding of the teaching session. The mutual perception of participants is rooted in their academic experiences and viewpoints accumulated over time.

However, recognizing these minor discrepancies prompts us to consider suggestions for enhancing everyone’s experience to its fullest potential.

Regarding *Preparation*, previous research shows that students’ decisions to prepare for teaching sessions result from a complex interplay of intrinsic factors, such as individual learning preferences, and extrinsic influences, including expectations from peers and educators [24]. The latter is in alignment with the principle in medical education that *assessment drives learning*, as the format may significantly influence students’ priorities and study behaviors [33].

Digital tools can enhance self-directed learning by promoting individual responsibility. E-learning modules effectively complement traditional methods and can be used as mandatory course components, offering certification upon completion.

*Participation by Schedule* remains a well-documented challenge in clinical teaching. Prior studies emphasize insufficient institutional support for clinical teachers, including limited time allocation, inadequate preparation for teaching roles, and restricted access to professional development opportunities [32]. Proposed solutions include protected teaching time, defined educator roles, and greater institutional recognition of teaching alongside research and patient care. Recent developments, such as specialized training programs and dedicated institutes for medical education, indicate that sustainable academic careers in clinical teaching are increasingly feasible [34]. Our findings emphasize that cultivating a supportive environment and a well-defined structure is critical for clinical educators to thrive.

With regard to *Understandable Explanations*, teachers in our study tended to rate their explanatory clarity lower than students, who were generally satisfied with the instruction. This discrepancy underscores the value of structured feedback mechanisms, which can both reassure educators and support ongoing improvement of teaching effectiveness.

## Conclusion

Our study on perceptions of students and instructors in ENT practical sessions revealed a strong alignment between the two groups. Improving the efficiency of practical medical education requires addressing discrepancies in preparatory expectations between medical students and clinical teachers, facilitating unhindered engagement for the latter, and embracing a mutual clarity of teaching delivery within the educational interface.

## Supplementary Information

Below is the link to the electronic supplementary material.

Supplementary Material 1.

Supplementary Material 2.

## Data Availability

The datasets analyzed during the current study are available from the corresponding author upon reasonable request. Descriptive data of the used questionnaire is provided in the manuscript.
