# Supplementary material for: A comparative study examining the perspectives of both students and clinical teachers during practical teaching sessions in otorhinolaryngology
Source: BMC Res Notes. 2026 Apr 27;19:185. doi: 10.1186/s13104-026-07802-w (PMC13112793; doi:10.1186/s13104-026-07802-w)
Supplement: Supplementary file 1 — Supplementary Material 1. [file 13104_2026_7802_MOESM1_ESM.pdf]

Teachingsession: (1-7)

Student: male ☐ female ☐ diverse ☐

Questionnaire ENT - Student

<u>Overall</u>

	Total disagree					Total agree
	1	2	3	4	5	6
1. The course was structured in a clear way.	☐	☐	☐	☐	☐	☐
2. The teaching materials (demonstration materials & instruments) supported my learning.	☐	☐	☐	☐	☐	☐
3. The course duration was appropriate.	☐	☐	☐	☐	☐	☐
4. The learning atmosphere was pleasant.	☐	☐	☐	☐	☐	☐
5. My prior knowledge for the course was adequate.	☐	☐	☐	☐	☐	☐
6. I prepared myself for the course.	☐	☐	☐	☐	☐	☐
7. The facilities were adequate.	☐	☐	☐	☐	☐	☐
8. The schedule allowed me to attend the course without stress.	☐	☐	☐	☐	☐	☐
9. My peers (tutors) supported my learning progress.	☐	☐	☐	☐	☐	☐

<u>Teacher</u>

10. The teacher explained the content in an understandable way.	☐	☐	☐	☐	☐	☐
11. The teacher supported my learning advancement.	☐	☐	☐	☐	☐	☐
12. The teacher was motivated.	☐	☐	☐	☐	☐	☐
13. The teacher was present throughout the entire session.	☐	☐	☐	☐	☐	☐
14. The teacher encouraged me to ask questions.	☐	☐	☐	☐	☐	☐

<u>Student</u>

15. The course motivated me.	☐	☐	☐	☐	☐	☐
16. The topic of the course interested me.	☐	☐	☐	☐	☐	☐
17. I participated actively.	☐	☐	☐	☐	☐	☐
18. The course increased my interest in ENT.	☐	☐	☐	☐	☐	☐

Practical skills

	Total disagree					Total agree
	1	2	3	4	5	6
19. I received feedback during/after the practical skills training.	☐	☐	☐	☐	☐	☐
20. I would feel confident applying the learned practical skills under supervision in clinical practice.	☐	☐	☐	☐	☐	☐

Interaction

21. I was able to interact well with the teacher.	☐	☐	☐	☐	☐	☐

Overall evaluation of the course:

What overall grade would you give this seminar?

(0 = insufficient; 3 = poor; 6 = sufficient; 9 = satisfactory; 12 = good; 15 = very good)

0	1	2	3	4	5	6	7	8	9	10	11	12	13	14	15
☐	☐	☐	☐	☐	☐	☐	☐	☐	☐	☐	☐	☐	☐	☐	☐

Additional remarks:

___

___

___
