# Supplementary material for: A comparative study examining the perspectives of both students and clinical teachers during practical teaching sessions in otorhinolaryngology
Source: BMC Res Notes. 2026 Apr 27;19:185. doi: 10.1186/s13104-026-07802-w (PMC13112793; doi:10.1186/s13104-026-07802-w)
Supplement: Supplementary file 2 — Supplementary Material 2. [file 13104_2026_7802_MOESM2_ESM.pdf]

Teachingsession: (1-7)

Teacher: male ☐ female ☐ diverse ☐

Questionnaire ENT - Teacher

Overall

	Total disagree					Total agree
	1	2	3	4	5	6
1. The course was structured in a clear way.	☐	☐	☐	☐	☐	☐
2. The teaching materials (demonstration materials and instruments) were adequate for the course.	☐	☐	☐	☐	☐	☐
3. The course duration was appropriate.	☐	☐	☐	☐	☐	☐
4. The course atmosphere was pleasant.	☐	☐	☐	☐	☐	☐
5. The students' prior knowledge was adequate.	☐	☐	☐	☐	☐	☐
6. I was prepared for the course.	☐	☐	☐	☐	☐	☐
7. The facilities were adequate.	☐	☐	☐	☐	☐	☐
8. The daily schedule allowed me to carry out the course without stress.	☐	☐	☐	☐	☐	☐
9. The peers (tutors) supported me in carrying out the course.	☐	☐	☐	☐	☐	☐

Teacher

10. I taught the content effectively.	☐	☐	☐	☐	☐	☐
11. The students achieved a learning advancement through my teaching session.	☐	☐	☐	☐	☐	☐
12. I was motivated.	☐	☐	☐	☐	☐	☐
13. I was present throughout the entire course.	☐	☐	☐	☐	☐	☐
14. I encouraged the students to ask questions.	☐	☐	☐	☐	☐	☐

Student

15. The students were motivated.	☐	☐	☐	☐	☐	☐
16. The topic of the course interested the students.	☐	☐	☐	☐	☐	☐
17. The students participated actively.	☐	☐	☐	☐	☐	☐
18. Through the course, I was able to increase the students' interest in ENT.	☐	☐	☐	☐	☐	☐

Practical skills

	Total disagree					Total agree
	1	2	3	4	5	6
19. I provided feedback during/after the practical skills training.	☐	☐	☐	☐	☐	☐
20. As a result of the course, the students are able to apply the practical skills under supervision in clinical practice.	☐	☐	☐	☐	☐	☐

Interaction

21. I interacted effectively with the students.	☐	☐	☐	☐	☐	☐

Overall evaluation of the course:
What overall grade would you give this seminar?
(0 = insufficient; 3 = poor; 6 = sufficient; 9 = satisfactory; 12 = good; 15 = very good)

0	1	2	3	4	5	6	7	8	9	10	11	12	13	14	15
☐	☐	☐	☐	☐	☐	☐	☐	☐	☐	☐	☐	☐	☐	☐	☐

Additional remarks:

___

___

___
